# Single-crystalline dendritic bimetallic and multimetallic nanocubes†
†Electronic supplementary information (ESI) available: Experimental details, supplementary TEM, SEM, STEM, XPS figures and electrochemical measurements. See DOI: 10.1039/c5sc01947h


**DOI:** 10.1039/c5sc01947h

**Published:** 2015-09-09

**Authors:** Yun Kuang, Ying Zhang, Zhao Cai, Guang Feng, Yingying Jiang, Chuanhong Jin, Jun Luo, Xiaoming Sun

**Affiliations:** a State Key Laboratory of Chemical Resource Engineering , Beijing University of Chemical Technology , Beijing 100029 , China . Email: sunxm@mail.buct.edu.cn; b Center of Electron Microscopy , Department of Materials Science and Engineering , Zhejiang University , Hangzhou 310027 , Zhejiang , P. R. China; c Center for Electron Microscopy , Institute for New Energy Materials & Low-Carbon Technologies , Tianjin University of Technology , Tianjin 300384 , China

## Abstract

Developing facial synthetic routes for fabrication of multimetallic nanocatalysts with open porous morphology, tunable composition and tailored crystalline structure is a big challenge for fabrication of low-cost electrocatalysts. Here we report on the synthesis of single-crystalline dendritic bimetallic and multimetallic nanocubes *via* a solvothermal co-reduction method. These cubes show highly porous, complex 3D inner connections but single-crystalline structure. Tuning the reduction kinetics of metal precursors and introducing galvanic reaction at the active sites during growth were believed to be the keys for the formation of such unique nanostructure. Electro-catalytic oxygen reduction (ORR) and methanol oxidation (MOR) on these catalysts showed dramatic enhancements for both cathodic and anodic electrocatalysis in fuel cells, which were attributed to their unique morphology and crystalline structure, as well as synergetic effect of the multi-metallic components. This work uncovers the formation mechanism of such complex single-crystalline dendritic multimetallic nanocrystals and offers a promising synthetic strategy for geometric and crystalline control of multimetallic nanocrystals with tailored physical and chemical properties, which will benefit the development of clean energy.

## Abstract 2

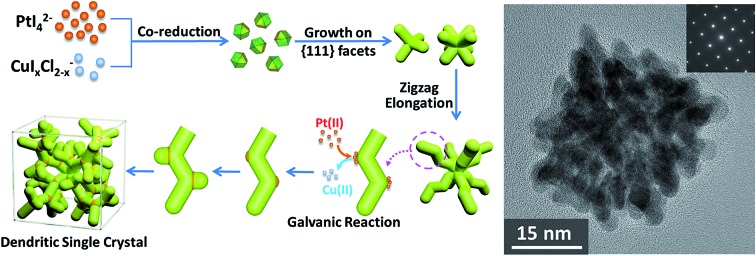
Single-crystalline highly porous nanocubes with complex 3D dendritic structure, uniform cubic morphology, and tunable bimetallic and multimetallic compositions were prepared by tailoring the growth kinetics in one-pot synthesis.

## Introduction

The sluggish kinetics of catalytic reactions (*e.g.* cathodic oxygen reduction reaction (ORR) and anodic methanol oxidation reaction (MOR)) and the high usage and cost of Pt catalyst are two main key obstacles for the commercial viability of proton exchange membrane fuel cells (PEMFCs), especially for transportation applications.^1^,^2^ Therefore, it is highly desirable to develop effective strategies for preparing electrocatalysts with largely reduced content of Pt while still showing superior performance.^3^–^8^

Bimetallic and multimetallic alloy nanostructures have broadly been believed to be substitutes for Pt catalysts due to their reduced use of Pt metal and enhanced overall catalytic performance.^1^,^3^,^5^,^6^,^9^–^22^ However, complex structure controls for nanoalloys are to date rarely reported.^23^–^26^ Ideally electrocatalysts should not only have three-dimensional (3D) open porous skeletons with controlled alloy compositions and morphologies, but also have tailorable crystalline structures.^1^

Facet-controlled synthesis of alloyed nanostructures with well controlled composition and crystalline structure has shown great potential for functional directed fabrication of electrocatalysts,^27^–^32^ but this strategy could only just be applied to some polyhedron nanoparticles instead of open porous nanostructures so far. Dealloying bi- or tri-metallic foils has been demonstrated an efficient way to obtain such nanoporous catalysts (*e.g.* nanoporous gold or platinum),^33^–^36^ but to gain single-crystalline nanoporous metals with specific facets exposure remains a challenge in spite of a few reports on dealloying synthesis of some porous low-dimensional nanoparticles so far.^37^,^38^ Recent reported dendritic nanostructures have shown capability to achieve open porous morphology and limited control over composition and crystalline structure,^10^,^24^,^39^–^42^ but the formation mechanism of such complex nanostructures was ambiguous and thus there still exists many limitations in controlled synthesis. Therefore, exploring new nanoalloy growth mechanisms and developing facial synthetic routes for fabrication of multimetallic nanostructures with open porous morphology and tailored crystalline structure is a big challenge with urgent priority.

Herein, single-crystalline highly porous nanocubes with complex 3D dendritic structure, uniform cubic morphology, and tunable bimetallic and multimetallic compositions were prepared by tailoring the growth kinetics in a one-pot synthesis. Tuning the reduction kinetics of metal precursors and tailoring galvanic reaction (*i.e.* oxidative etching, between noble metal ion and reduced non-noble metal) at the active sites during growth were believed to be the keys for the formation of such unique nanostructures. With 3D open porous networks, ultrathin branches, controllable alloyed composition and tailored crystalline structure, such single-crystalline dendritic cubes showed superior ORR and MOR electrocatalytic performance and ultrahigh durability, which are almost 10 times higher than that of state-of-art Pt/C catalysts.

## Results and discussion

In a typical synthesis of single-crystalline dendritic bimetallic PtCu nanostructures, polyvinylpyrrolidone (PVP, K30, 100 mg) and KI (1.0 mmol) were dissolved under vigorous stirring in *N*,*N*-dimethylacetamide (DMAC, 3 mL) and then mixed with 2 mL of an aqueous solution containing K_2_PtCl_4_ and CuCl_2_ (total amount of 0.2 mmol but with different molar ratios) in a 10 mL Teflon-lined stainless steel autoclave. The autoclave was maintained at 150 °C for 4 h, and then cooled down to room temperature. The black precipitate was washed with ethanol and acetone several times and dispersed in ethanol for use.

**Fig. 1:**
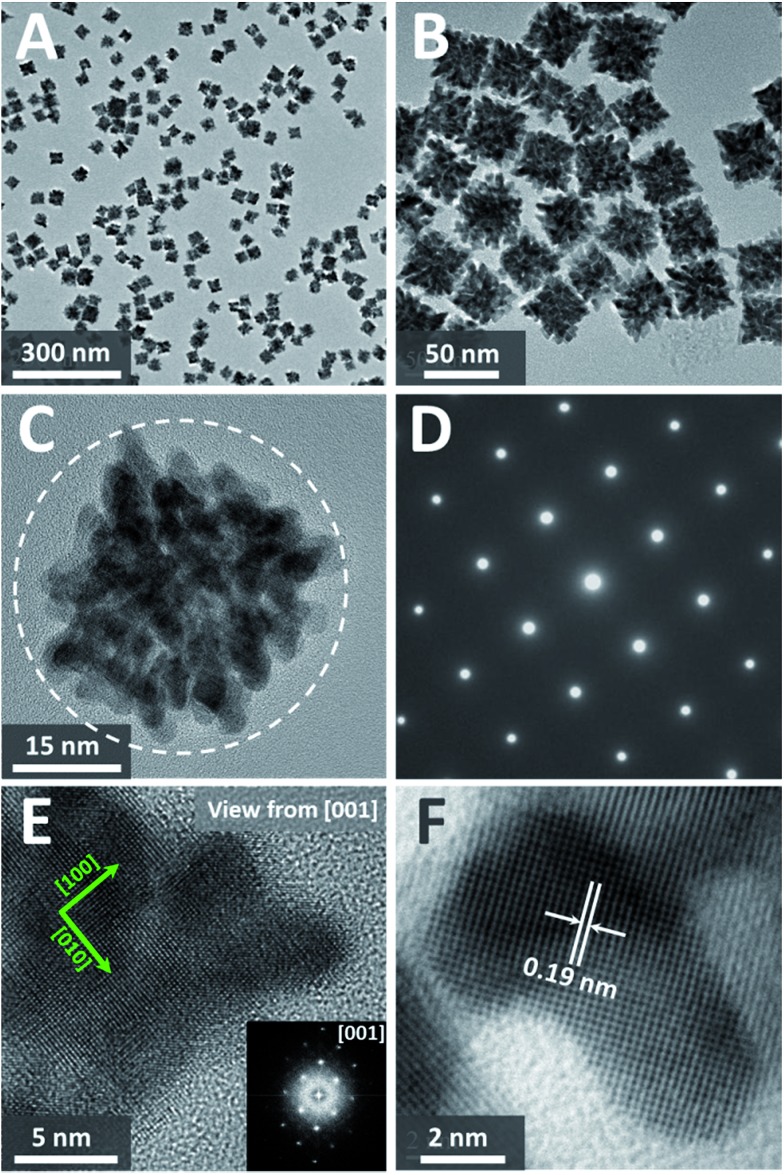
(A) and (B) TEM images of dendritic Pt_3_Cu cubes. (C) HRTEM and (D) corresponding electron diffraction (ED) pattern of a typical single-crystalline dendritic Pt_3_Cu cube. (E) Enlarged HRTEM image and corresponding FFT pattern of a corner of the single-crystalline dendritic Pt_3_Cu cube. (F) Further enlarged HRTEM of a branch of the dendritic Pt_3_Cu cube.

Transmission electron microscopy (TEM) image (Fig. 1A) shows that the product consists of uniform dendritic nanocubes with a yield approaching 100%. The dendritic nanocubes are highly monodisperse, and the size of the nanocrystals lies in a narrow range from 32 to 42 nm, with an average diameter of 36 nm (Fig. 1B). Enlarged TEM image of a typical dendritic cube (Fig. 1C) shows that nanocubes have porous nanostructure with highly multi-branched subunits, and the branch diameter in the dendritic PtCu nanocubes is measured to be as thin as 3.7 nm (Fig. 1F). It is shown that the branches are connected to each other in an orderly manner to form a hierarchical cubic structure, and each cube consists of tens of branches, making the structure highly porous. The diameter of the pores between the branches is about 2–5 nm, which could provide reactant pathways in catalytic reactions. To our surprise, such porous dendritic cubes show single-crystalline feature, as evidenced by the electron diffraction (ED) pattern of a single cube (Fig. 1D). Viewing from the [001] direction of a cube, high-resolution transmission electron microscopy (HRTEM) image and corresponding fast-Fourier transformation (FFT) pattern confirmed (200) crystalline faces dominated the single-crystalline cubic structure (Fig. 1E). As shown in Fig. 1F, the observed *d*-spacing (0.19 nm) in the branch region corresponded to the (200) planes of the fcc Pt_3_Cu structure. It should be noted that many atomic steps/corners are exposed on the branch surface, similar to those observed on nanoporous gold,^33^ which could act as highly catalytic sites.

**Fig. 2:**
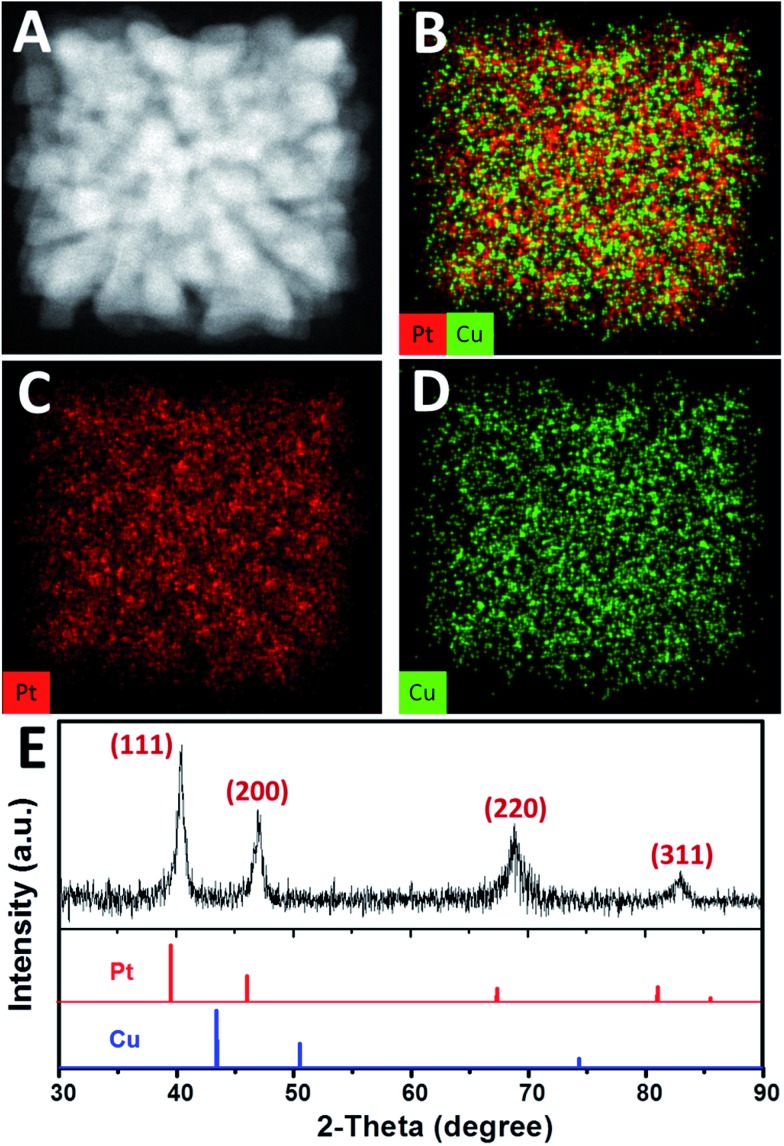
(A) HAADF-STEM image and (B)–(D) EDX-mapping of a single-crystalline dendritic Pt_3_Cu cube. (E) XRD pattern of the single-crystalline dendritic Pt_3_Cu cubes. The standard diffraction peaks for Pt (JCPDS 03-065-2868) and Cu (JCPDS 03-065-9743) metals are also shown for comparison.

High-angle annular dark field-scanning transmission electron microscopy (HAADF-STEM) image (Fig. 2A) further confirmed the single-crystalline dendritic Pt_3_Cu cubes have open porous structure but very complex inner networks. To identify the chemical compositions, typical obtained colloidal nanocrystals were analysed using energy-dispersive X-ray spectroscopy (EDX) of ChemiSTEM™ of an aberration-corrected FEI Titan S/TEM instrument (see details in ESI†) and inductively coupled plasma optical emission spectrometry (ICP-OES). EDX-mapping of the dendritic cubes (Fig. 2B–D) shows that Pt and Cu atoms are uniformly distributed in each branch, demonstrating the alloyed structure. However, Pt mapping exhibited higher contrast than Cu and showed several dots with high brightness, possibly because there are some Pt rich regions. Elemental analysis suggests an atomic ratio of ∼3 : 1 (72.56% : 27.44%) of the cubes, which coincided with ICP-AES results (72.75% : 27.25%), demonstrating the compositional homogeneity of the dendritic cubes.

The X-ray diffraction (XRD, Fig. 2E) pattern of the dendritic Pt_3_Cu cubes displays characteristic peaks that are in agreement with those of well crystalized Pt pattern with face-centered-cubic (fcc) structure (JCPDS no. 03-065-2868) but shift to higher angle (*i.e.* shift to Cu peak), confirming an alloyed structure. The shift of the {111} peak from 39.7° (pure Pt) to 40.5° was consistent with the calculated value for well crystallized Pt_3_Cu. No detectable impurity peaks of pure Pt and/or pure Cu were observed in the XRD pattern, indicating that only a single Pt_3_Cu phase exists in the sample. The intensity of the {200} peak is higher than standard fcc crystals, benefitting from the cube-like external morphology and apparently [100] preferred orientation of the whole cube, which was consistent with the phenomenon that more than 80% of particles appearing square under TEM observation. Besides, the XRD peaks are broad, demonstrating ultrasmall crystal size of the final products. As calculated using the Scherrer equation on the {220} diffraction peak, the grain size is about 3 nm, which was consistent with the width of branches observed in the HRTEM image (Fig. 1F), suggesting (110) facets exposure of the inner branches. Additionally, a Cs-corrected HRTEM image of an intermediate showed typical (110) facet atom arrangement (Fig. S2A†) of the branch surfaces and electrochemical cyclic voltammetry in the succeeding figure further confirmed (110) facet exposure.

**Fig. 3:**
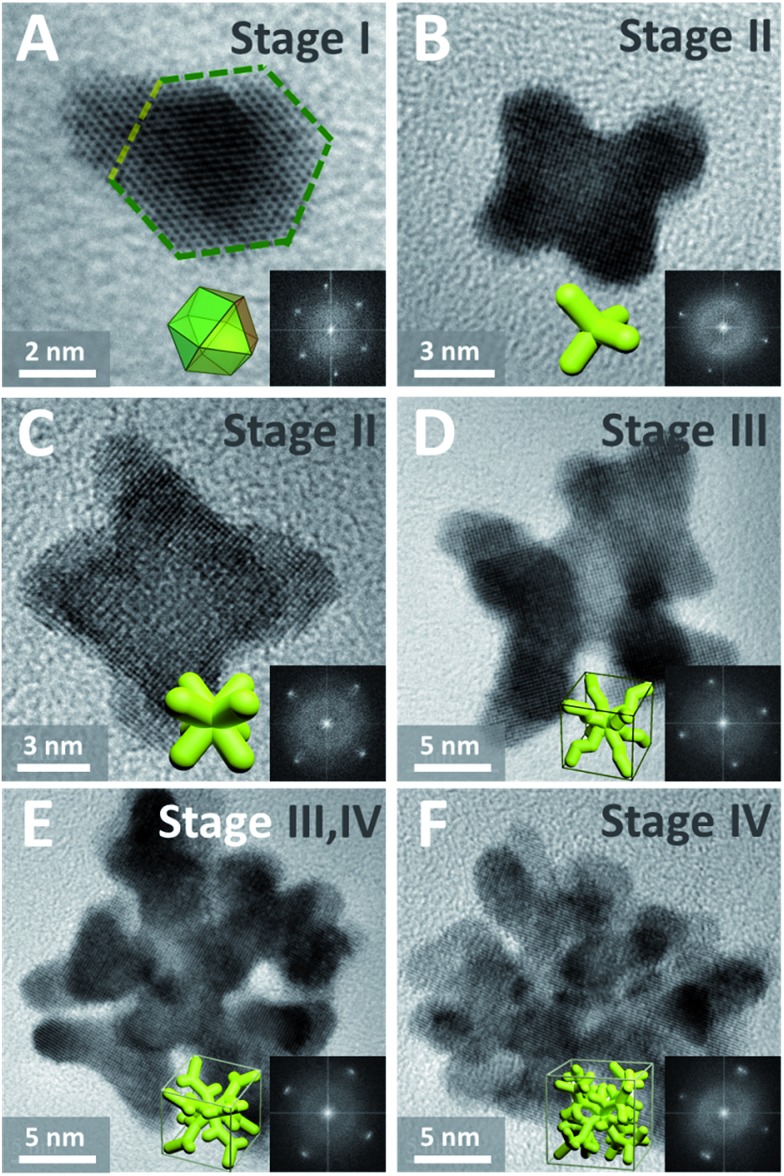
(A)–(F) HRTEM images (insets show corresponding FFT patterns and idealized representations) of dendritic Pt_3_Cu intermediates formed at different reaction stages.

Such complex dendritic single crystals with well-defined external morphology are quite different from traditional single crystals, as the dendritic single crystals show no flat crystalline planes but rather complex ordered inner networks. Therefore, we believe there should be a specific growth pathway for such porous single crystals. Thus time-dependent structure evolution reaction was carried out to uncover the formation process of the single-crystalline dendritic cubes, as shown in Fig. 3 (also see Fig. S1† for reaction time slots). Morphology evolution clearly shows four stages of the formation procedure:

 

(I) Formation of primary polyhedral seeds (Fig. 3A).

(II) Stacking on specific facets ({111} facets) of the seeds to form tetrapods and octapods (Fig. 3B and C).

(III) Elongation of the octapods (Fig. 3C–E).

(IV) Formation of new branches (Fig. 3E and F).

It should be noted that the last two stages are not separate; formation of new branches always occurs along with elongation of the skeleton.

In order to further investigate the growth behavior of the dendritic single-crystalline alloy, HRTEM was used to analyze the crystalline structure of the intermediates formed at different reaction stages. It was found that all the intermediates are single crystals but exposed different crystalline facets. At the initial stage, the formed primary seeds exposed (111) facets (Fig. 3A), while all the following intermediates show (100) facets (Fig. 3B–F). This was coincident with the previous study on the formation of Pt octapods reported by Xiong *et al.*^43^ The primary particles are single-crystal cuboctahedral seeds that are enclosed by six (100) facets and eight (111) facets. It is reported that the halogen ions in the synthetic environment had a significant capping effect on Pt(100) faces,^43^ thus the six (100) facets are protected from atomic addition in the formation of octapods. Besides, the concentration of halogen ions in this system is 10 times higher than metal precursor; therefore, cuboctahedral seeds can gradually grow into tetrapods and then octapods when freshly formed atoms are added to the (111) corners (Fig. 3A) and thus the final products showed eight main branches pointing to the eight corners of the cube.

The growth behaviour of PtCu nanocrystals at initial reaction stages were similar to that of single-element Pt octapods through reduction control using I^–^ ions, which could tune the reduction kinetics of the two precursors by coordinating with Pt^II^ to form a more stable PtI_4_^2–^ (PtI_4_^2–^/Pt, *E* = 0.32 V; PtCl_4_^2–^/Pt, *E* = 0.758 V) and at the same time reducing Cu^II^ to Cu^I^ species, which is easily further reduced to Cu^0^ (Cu^+^/Cu, *E* = 0.52 V, Cu^2+^/Cu, *E* = 0.34 V).^44^ Therefore the reduction of Cu^II^ and Pt^II^ occurred almost simultaneously and thus the growth model of such an alloy was almost the same as single Pt crystals at the initial stages (stages I and II).

However, different from the formation of Pt octapods, such PtCu octapods could further grow new branches to form a single-crystal dendritic cube in the later reaction stage. Generally, there are two possible mechanisms to understand the formation process of branched dendritic structures: one is oxidative etching during the crystal growth;^45^ in which oxidant (such as O_2_) could oxidize the reduced metal atoms back to ions while halogen ions could help coordinate and stabilize the newly formed metal ions, therefore, competition of reduction and oxidation could result in dendritic structure. The other way is oriented attachment of primary particles;^46^,^47^ in which the nuclei or primary seeds act as precursors of particles. Through oriented attachment, the primary particles unite to each other when surface lattices match and finally result in networks or dendrites. The difference between these two mechanisms is reflected in the intermediate and we believe the formation of such single-crystalline dendritic alloyed cubes was the result of oxidation etching, for the reasons indicated below.

For oriented attachment, in order to provide enough “precursors” for attachment, there must be a huge amount of primary particles in the initial nucleation stage or there should exist primary particles during the formation of dendritic structure; however, we could only obtain few primary seeds in the nucleation stage, even when centrifuged at 90 000 rpm (694 000*g*, Beckman L-100XP) for hours. Besides, there did not exist any tiny primary particles in the intermediate we captured in the 30–45 min reaction time slot.

Another obvious difference between the two mechanisms is the composition deviation during the formation process. For oriented attachment, the Pt/Cu ratio of the intermediate should be constant since the primary particles should have the same composition. However, for oxidative etching, galvanic reaction between the formed alloy and environmental noble metal ions would cause composition deviation.

To experimentally confirm the hypothesis, we monitored the composition variation of the intermediate based on ICP results, as shown in Table 1. It was found that the Pt/Cu ratios of early intermediates were quite high, probably because the precursor concentration was much higher than the nanoparticle concentration at early stages, and thus galvanic reaction between Pt^2+^ and Cu^0^ was substantial. However, with the dendritic cubes growth, the Pt/Cu ratio decreased due to low noble metal ion concentration and strong reduction ability of DMAC solvent. This trend was also confirmed by EDX elemental analysis of a single intermediate (45 min sample, Pt : Cu = 3.07 : 1, Fig. S2†) and a final dendritic cube (8 h, Pt : Cu = 2.64 : 1, Fig. 2), suggesting that the formation of such single-crystalline dendritic cubes follows atomic growth along with oxidative etching.

**Table 1:** Composition variation of the intermediates

Time/min	Pt : Cu
30	4.23 : 1
33	4.02 : 1
37	4.00 : 1
40	3.94 : 1
45	3.14 : 1
60	2.89 : 1
120	2.80 : 1
480	2.67 : 1

In the elongation of octapod skeleton stage (Fig. 3D and S1D†), it could be seen that each branch grew in a zigzag shape, this was because the Cu atoms were “defect” atoms in the Pt lattice and they would induce some twin boundaries in the elongation of the branches by decreasing the formation energies of twin boundaries.^40^,^45^,^48^ This phenomenon coincided with our previous study on the formation of dendritic pyramid caps.^44^,^49^ On the convex sites of the zigzag branch, there are more step/corner atoms and thus these sites are more active for the growth of new branches *via* galvanic reaction. In order to evidence that the galvanic reaction indeed triggered the formation of new branches we monitored the element distributions on a dendrite.

**Fig. 4:**
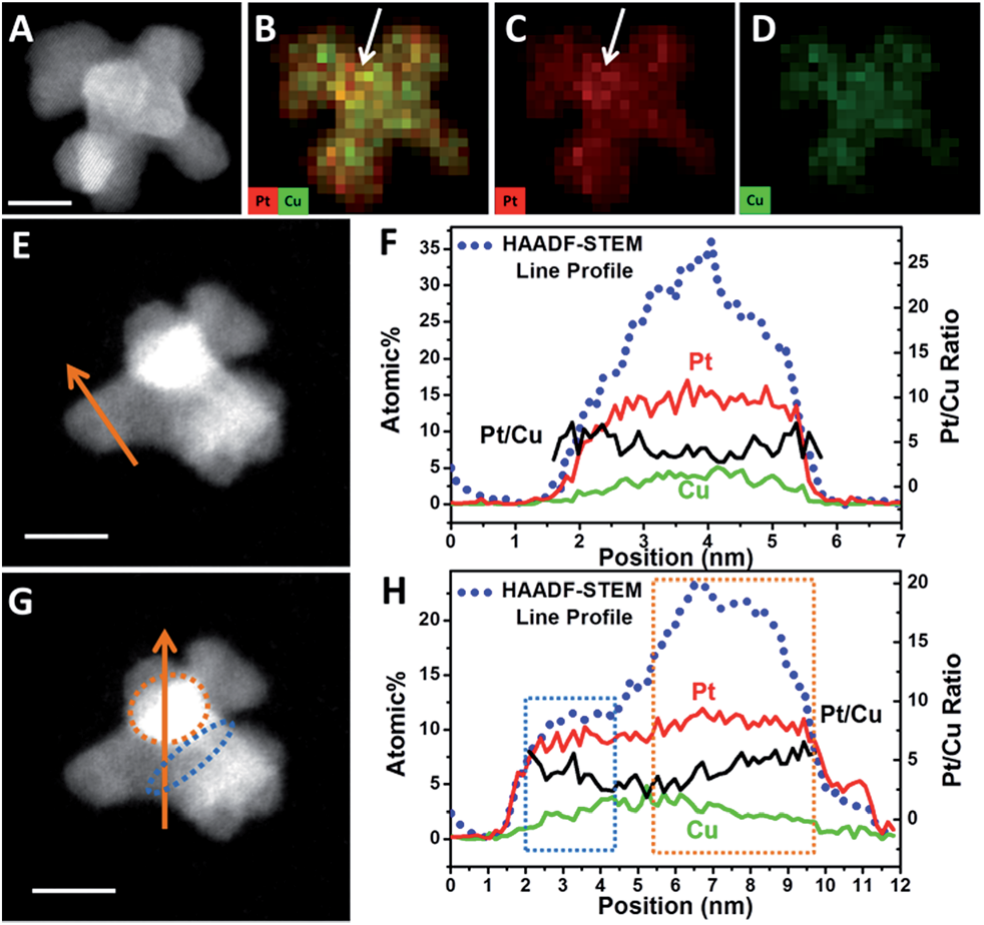
(A) HAADF-STEM image and (B)–(D) quantified display of EDX-mapping of an octapod intermediate. (E) and (G) HAADF-STEM image and line scanning across a branch or branch connecting sites. (F) and (H) HAADF-STEM line profiles (blue dots), elemental distribution of Pt (red lines) and Cu (green line) and Pt/Cu ratios (black lines) along the line scanning region marked in (E) and (G). Scale bars are 5 nm.

HAADF-STEM and quantified display of EDX-mapping images of a PtCu intermediate (Fig. 4A–D) show that the Pt and Cu are well dispersed in all the branches. However, at the convex sites, the Pt atoms are more concentrated compared with Cu, as marked in Fig. 4B. It should be noted that the quantified display has already eliminated the specimen thickness effect and background effect. STEM-line profiles also show two different regions, across the branch and the connection sites of branches. Across the branch, the STEM image is relatively dim (Fig. 4E) and the continuous signals of the line profile shows a typical cylinder shape of the branch (Fig. 4F); while at the connecting site, the image is quite bright (Fig. 4G) and the line profile shows a changed region as boxed in Fig. 4G and H. One reason is that STEM is thickness sensitive and the connecting site is thicker than the branch. Another reason is that there are heavy elements (*i.e.* Pt) aggregated at the connection sites (STEM is sensitive to atomic number (*i.e. Z* contrast)).

In order to reveal the hypothesis, element distribution was applied along the line profile region (Fig. 4F and H). Across the branch, the variation of Pt and Cu distribution are almost the same and the Pt/Cu ratio changes slightly from the edge to the centre of a branch (Fig. 4F), though the concentration of Pt was a little higher on the edges than in the centre. However, when the line profile reached the branch connecting sites (boxed regions), Pt and Cu concentration changed differently: when scanning from a branch connecting site (blue dots region in Fig. 4G) to the centre of a branch, the Pt/Cu ratio decreased gradually (Fig. 4H, blue box); while scanning from the centre of the branch to another branch connecting site (orange dots region in Fig. 4G), the atomic percentage of Pt increased slightly while Cu decreased more substantially and the Pt/Cu ratio increased gradually (Fig. 4H, orange box), demonstrating that most of the Cu atoms at the branch connecting sites were replaced by Pt, confirming the key role of galvanic reaction between Pt^2+^ and Cu^0^ in the formation of new branches.

Control experiments also confirmed the galvanic reaction. Changing the Pt precursor from Pt^2+^ to Pt^4+^, the resultant products were also dendritic cubes (Fig. S3A and B†), but when we changed the Pt precursor from ionic K_2_PtCl_4_ to covalent Pt(acac)_2_, the products were only some small nanoparticles (Fig. S3C†), demonstrating that the dendritic structure is related to ionic Pt precursors. When we changed the Pt^2+^/Cu^2+^ molar ratio from 19 : 1 to 1 : 3, only when this ratio was larger than 1 did dendritic structures result (Fig. S4A–D†); less Pt^2+^ would greatly reduce the amount of branches (Fig. S4E and F†). This further confirmed the close relation between galvanic reaction and dendritic structures.

**Scheme 1:**
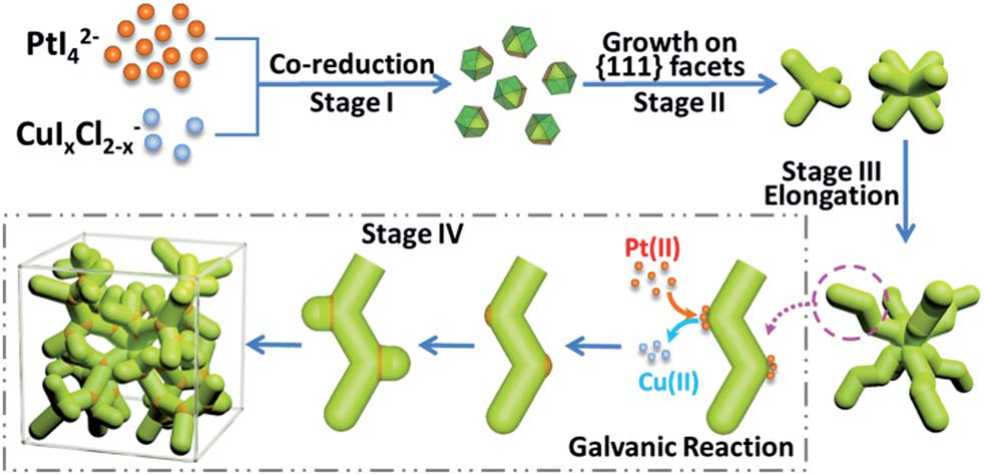
Schematic formation of single-crystalline dendritic cubes.

Based on the above results, a formation mechanism was proposed, as shown in Scheme 1. The metal precursors were first co-reduced to form cuboctahedral seeds, then I^–^ ions controlled selective growth on {111} facets, which resulted in tetrapods and further grew into octapods. Further elongation of the octapods resulted in primary branches with “zigzag” shape, which was induced by “defects” (Cu atoms in Pt lattice).^44^,^50^ After that, galvanic reaction between Pt(ii) and Cu(0) on the relatively more active convex sites induced the growth of new branches and repeated growth of new branches would finally create the single-crystalline dendritic cubes.

The relatively complex formation procedure made the structure very sensitive to the synthetic parameters. Besides the Pt/Cu precursor ratio and metal precursor species mentioned above, variations of coordination reagents (Fig. S5†), reaction temperature (Fig. S6†) and solvent species all would weaken the dendritic structure or even eliminate the branched structures due to the change of reduction kinetics of the two precursors and oxidative etching process.

**Fig. 5:**
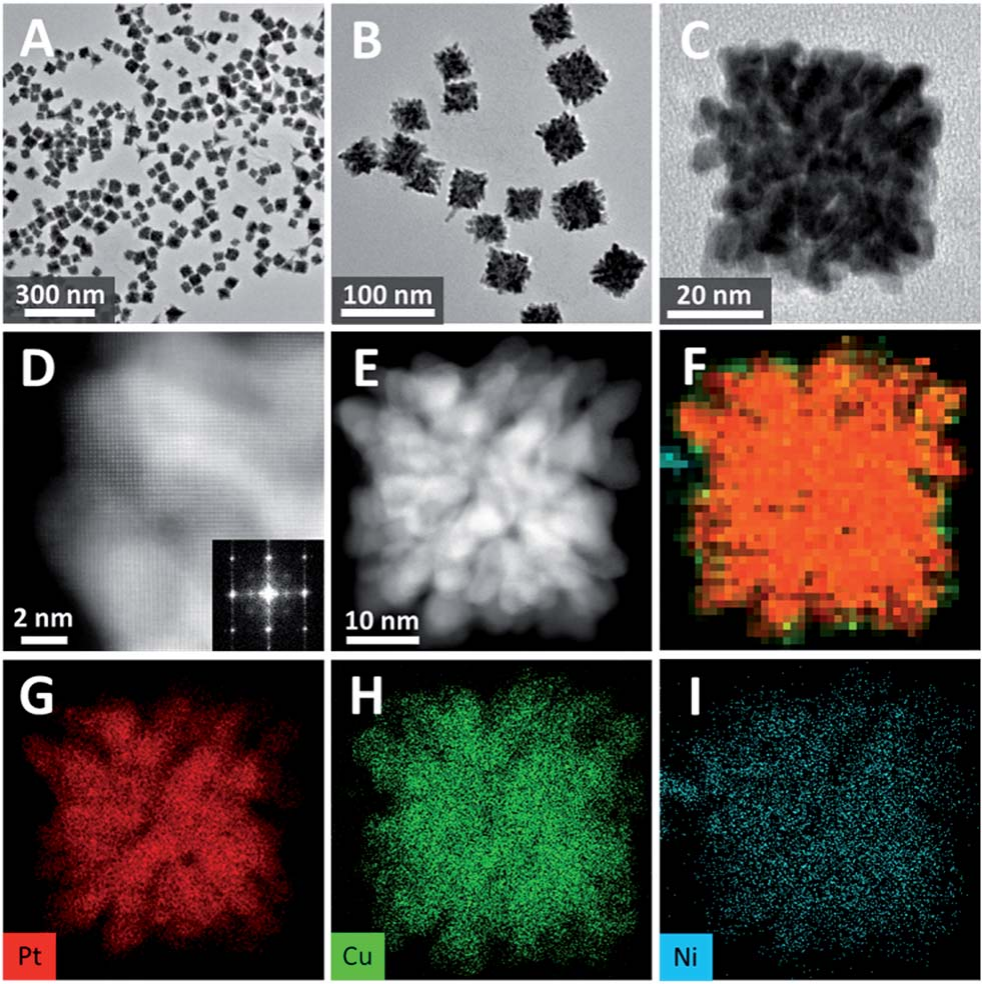
(A)–(C) TEM images of dendritic PtCuNi cubes. (D) STEM and corresponding FFT pattern of a corner of a single-crystalline dendritic PtCuNi cube. (E) HAADF-STEM image and (F)–(I) EDX-mapping of a single-crystalline dendritic PtCuNi cube.

Though bimetallic nanocrystals have led to great advances in various catalytic issues, multimetallic nanocrystals are even more promising candidates since the regularity of their electronic structures are more flexible and controllable. Therefore, we also tried to extend the synthesis strategy of bimetallic single-crystalline dendritic structures to multimetallic nanocrystals. As a typical example, when Ni^2+^ precursors were added, single-crystalline dendritic PtCuNi nanocubes was synthesized, as shown in Fig. 5.

TEM image shows that the product yield was also approaching 100% (Fig. 5A), and the average size of the PtCuNi nanocubes (42 nm) were a little larger than PtCu cubes (Fig. 5B). An enlarged TEM image of a typical dendritic PtCuNi cube (Fig. 5C) shows that the multimetallic dendritic cubes have more and denser branches than that of PtCu bimetallic dendritic nanocubes, possibly because the introduction of Ni atoms strengthened the galvanic reaction and thus formed more branches. STEM image and corresponding FFT pattern showed that the multimetallic dendritic cubes were also single crystals with exposed {200} facets (Fig. 5D), confirming the inheritance of crystalline structure from bimetallic to multimetallic dendritic nanocubes. HAADF-STEM image (Fig. 5E) shows that the inner channels of PtCuNi nanocubes were thinner than that of PtCu cubes, confirming that multimetallic dendritic cubes have denser branches. EDX-mapping of the dendritic cubes (Fig. 5F–I) shows alloyed structure with Pt, Cu and Ni atoms well dispersed in branches.

This strategy was also applicable to addition of other transition metals (*e.g.* Mo, Fig. S7†) but interestingly, Cu element was found the key to trigger the formation of such dendritic structure, even 5% atomic ratio of Cu addition would result in typical single-crystalline dendritic cubes as shown in Fig. S4A.† This was attributed to the fact that copper was the only element whose reduction potential could be tuned close to that of platinum, and thus alloy structure could form at the initial stage. Therefore, the copper atoms could induce zigzag growth pattern of the branch and also provide galvanic reaction sites for the formation of new branches. Since the usage of copper could be as low as 5%, it is possible to tune the compositions in a wide range and meanwhile retain the dendritic cubic morphology. As an example, when we changed the feeding ratio of PtCuNi system from 9 : 1 : 1 to 9 : 1 : 9, the composition varied with the same trend (Table S1†), demonstrating the flexibility of composition control. Besides, composition variation would not disturb the crystallinity, all the PtCuNi samples with different compositions showed single-crystal structures, as evidenced by the electron diffraction patterns (Fig. S8†).

With tailored crystalline structure and open porous but interconnected morphology, the as-prepared single-crystalline dendritic nanocubes should have a high surface area and excellent electroconductivity, and thus are expected to be ideal electrocatalysts. Thus methanol oxidation and oxygen reduction reaction were performed on the PtCu and PtCuNi nanocubes, respectively, to show the advantages of such catalysts in fuel cell application.

**Fig. 6:**
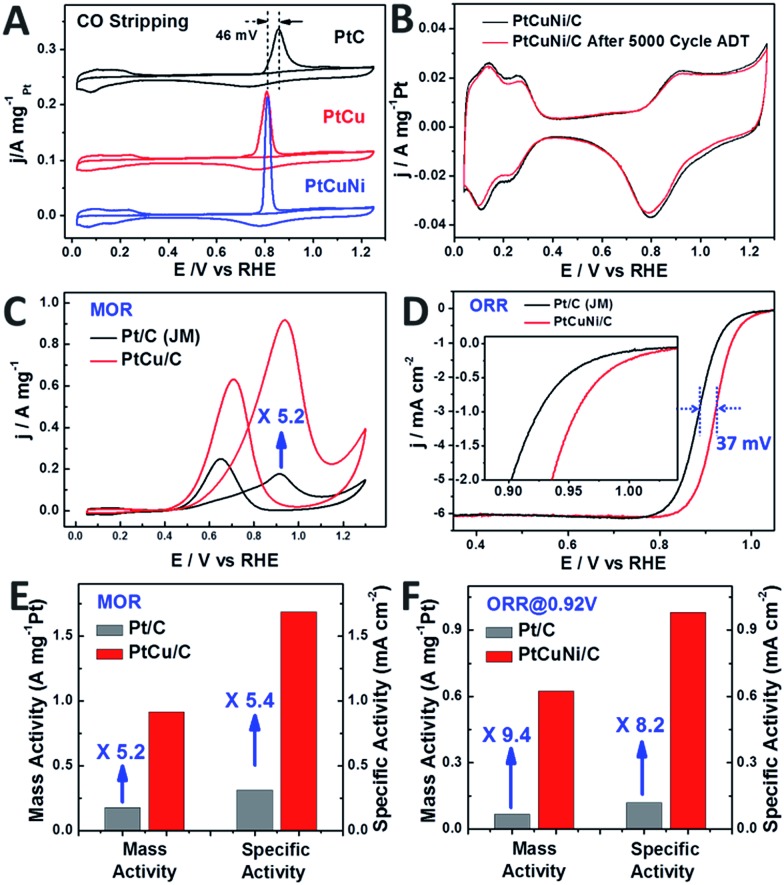
(A) Mass normalized CO stripping curves of Pt/C (Johnson Matthey, 20 wt%) and single-crystalline dendritic Pt_3_Cu and PtCuNi nanocubes in 0.5 M H_2_SO_4_ solution. (B) CV curves of PtCuNi dendritic cubes before and after 5000 cycles of oxygen reduction reaction (ORR) accelerated durability test (ADT). Only 2.7% activity loss was observed. (C) CV curves of Pt/C and dendritic Pt_3_Cu nanocubes in methanol oxidation, revealing a 5.2-fold enhancement on mass activity. (D) ORR polarization curves of PtCuNi/C and commercial Pt/C recorded at a rotation rate of 1600 rpm. The half-wave potential of dendritic PtCuNi nanocubes shifted 37 mV to higher potential than Pt/C. (E) and (F) Specific activity and mass activity comparison for methanol oxidation (PtCu/C and commercial Pt/C) and oxygen reduction reaction (PtCuNi/C and commercial Pt/C), which are given as kinetic current densities normalized to the ECSA and the loading amount of Pt, respectively.^51^ The Pt loading was 4 μg for all the catalysts in methanol oxidation reaction test and were 2 and 4 μg for nanodendrites and commercial Pt/C in oxygen reduction reaction test, respectively.

Fig. 6A compares the mass normalized CO stripping curves of these catalysts recorded in 0.5 M H_2_SO_4_ solution at a sweep rate of 20 mV s^–1^. The high CO desorption charge suggests that the dendritic PtCu and PtCuNi cubes have much higher electrochemically active surface area (ECSA) than commercial Pt/C. As calculated by measuring the charge collected in the CO desorption region after and assuming a value of 420 μC cm^–2^ for the adsorption of a CO monolayer, the ECSA of PtCu and PtCuNi cubes were 54.3 and 63.8 m^2^ g^–1^, respectively, which are comparable or higher than that of commercial Pt/C catalyst (56.7 m^2^ g^–1^). Moreover, the CO desorption peaks were much sharper than that of commercial Pt/C, revealing specific facet exposure of the single-crystalline dendritic cubes, as evidenced by the typical (110) facet CV curves in Fig. 6B.^9^ The combination of the highly accessible surface area and complex inner connections should endow the dendritic nanocrystals with superior electrocatalytic activity.

Previous study shows that alloying Cu into the Pt lattice could dramatically improve its methanol oxidation performance, especially on the {100} and {110} facets.^52^,^53^ Thus electrocatalytic oxidation of methanol was performed on the synthesized dendritic Pt_3_Cu cubes (Fig. 6C) and commercial Pt/C catalysts. It is revealed that the mass activity and specific activity of dendritic Pt_3_Cu cubes are 5.2 and 5.4 times that of commercial Pt/C (Fig. 6E). Such high electrocatalytic activity for the Pt_3_Cu cubes are ascribed to their structural and morphological superiorities compared with traditional Pt/C particles. Besides high current density, the dendritic Pt_3_Cu nanocubes also showed a high anti-CO poisoning performance, as evidenced by a much higher *I*_f_/*I*_b_ value and 46 mV negative shift of CO-stripping peak as compared to that of Pt/C, indicating a weaker Pt–CO bond strength on the Pt_3_Cu surface.

Researchers have reported that Pt_3_Ni alloys exhibit extremely high oxygen reduction reaction (ORR) activity that is 10-fold higher than pure Pt,^9^ thus we evaluated the electrocatalytic performance of the crystalline dendritic Pt_3.06_Cu_0.36_Ni_1_ nanocubes for ORR since this Pt/Ni ratio was closest to the best reported ones. Fig. 6D shows the ORR polarization curves for PtCuNi nanocubes and commercial Pt/C catalysts after IR correction. The half-wave potentials of porous PtCuNi nanocubes and commercial Pt/C are 0.921 and 0.884 V, respectively, indicating that the activity of the PtCuNi nanocubes is higher than that of commercial Pt/C. We calculated the kinetic currents from the ORR polarization curves by considering the mass-transport correction according to the Levich–Koutecky equation: 1/*i* = 1/*i*_k_ + 1/*i*_d_ (where *i*_k_ is the kinetic current and *i*_d_ is the diffusion-limiting current).^41^ The kinetic currents at 0.92 V were normalized with respect to both ECSA and the loading amount of metal Pt to compare the activity for different catalysts. As shown in Fig. 6F, both the mass activity and specific activity of the PtCuNi nanocubes were ∼10 times higher than that of the Pt/C catalyst, suggesting a super electroactivity, benefitting from the extremely high surface area, porosity, conductivity and electrocatalytic reactivity.

The bimetallic and multimetallic dendritic nanocubes showed composition dependent electrocatalytic performances. For both MOR and ORR reactions, the Pt_3_X composition exhibited the best performance, while either higher or lower Pt contents resulted in relatively poor electrocatalytic activities (Fig. S9†).

Moreover, the single-crystalline dendritic multimetallic nanocubes showed very high stability. Electrochemical durability test was performed using accelerated durability tests (ADT) by cycling the potential between 0.6 and 1.0 V (*vs.* RHE) in O_2_-saturated 0.1 M HClO_4_ at a scan rate of 50 mV s^–1^. As shown in Fig. 6B, the dendritic PtCuNi nanocubes show almost no change in CV curves after 5000 cycles ADT test. Calculated from the H adsorption/desorption region, the dendritic PtCuNi nanocubes only have 2.7% loss in ECSA. Besides, the ORR polarization curve only shifts 11 mV to negative potential (Fig. S8C†), possibly because of atomic rearrangement on the alloy surface to form a Pt-skin structure.^1^ However, 30.2% loss of ECSA (Fig. S10B†) and 39 mV negative shift of ORR polarization curve (Fig. S10D†) were observed for Pt/C catalyst after only 2500 cycles ADT test, revealing that the durability of dendritic multimetallic nanocubes is much better than commercial Pt/C. HRTEM images of the sample before and after ADT test showed no changes in morphology while XPS spectrum of Pt element showed only 0.1 eV difference before and after 5000 cycles ADT test (Fig. S11†), which is within the error range of XPS instrument, suggesting maintenance of the electronic structure.^42^ Besides, EDX of the PtCuNi nanodendrites after ADT test suggested a Pt/Cu/Ni ratio of 8.61 : 1 : 2.69, which is almost the same as the sample before ADT test (8.49 : 1 : 2.78), with a tiny increase of Pt content, possibly forming a Pt-skin structure after ADT test. The ultrahigh durability might be ascribed to the atomic mobility change after alloying three kinds of metals.^25^

## Conclusions

In summary, single-crystalline dendritic bimetallic and multimetallic nanocubes have been synthesized by good kinetic control of the co-reduction process of the precursors. Competition of reduction reaction and oxidative etching played key roles for such morphological and crystalline engineering. The as-synthesized dendritic Pt_3_Cu cubes showed excellent electrocatalytic activity and dramatic durability, demonstrating the great potential of dendritic alloy structure for constructing advanced electrocatalysts due to their tailored composition and crystalline structure, fully exposed active sites and integrated conductive structure. This result provides strategies and opportunities to design and fabricate other dendritic bimetallic structures with well controlled morphology and crystalline structure, which benefits the development of clean energy and green chemistry.

## Supplementary Material

Supplementary information
