# Supplementary material for: Single-crystalline dendritic bimetallic and multimetallic nanocubes
Source: Chem Sci. 2015 Sep 9;6(12):7122–9. doi: 10.1039/c5sc01947h (PMC5951113; doi:10.1039/c5sc01947h)
Supplement: Supplementary file 1 [file SC-006-C5SC01947H-s001.pdf]

Electronic Supplementary Material (ESI) for Chemical Science.
This journal is © The Royal Society of Chemistry 2015

Supporting Information for

Single-Crystalline Dendritic Bimetallic and Multimetallic Nanocubes

Yun Kuang, Ying Zhang, Zhao Cai, Guang Feng, Yingying Jiang, Chuanhong Jin, Jun Luo and Xiaoming Sun*

Experimental Details

Synthesis of dendritic PtCu nanocubes. In a typical synthesis, PVP (K30, 100 mg) and KI (1.0 mmol) were dissolved under violent stirring in N, N-dimethylacetamide (DMAC, 3 mL) at room temperature and then mixed with 2ml of aqueous solution containing K_2PtCl_4 and $CuCl_2$ (total amount of 0.2 mmol but with different molar ratios) in a 10mL Teflon-lined stainless steel autoclave. The autoclave was maintained at 150 °C for 4 h, and then cooled down to room temperature. The black precipitate was washed by ethanol and acetone for several times and dispersed in ethanol for use.

Synthesis of dendritic PtCuNi and PtCuMo nanocubes. The synthesis procedures were the same as PtCu but with varied Pt/Cu ratios and the addition of $NiCl_2$ (or Molybdenyl acetylacetonate $MoO_2(acac)_2$). The total amount of metal precursors is 0.2 mmol but with different molar ratios according to the samples required.

Materials Characterization. X-ray diffraction (XRD) patterns were collected on Shimadzu XRD-6000 with Cu Kα radiation (40 kV, 30 mA, λ = 1.5418Å), recorded with 2θ ranging from 30° to 90°. Elemental analysis was investigated by inductively coupled plasma optical emission spectrometry (ICP-OES; Thermo Scientific iCAP 6300). The morphologies of as-synthesized samples were characterized by transmission electron microscopy (TEM; FEI G^2 20) and high-resolution transmission electron microscopy (HRTEM; JEOL, JEM-2100, 200 kV). Scanning Transmission Electron Microscopy (STEM) measurements were performed at 200 kV by a cubed Titan Themis with a probe corrector, an image corrector and a ChemiSTEM™ system of EDX detectors. Complete structural and elemental information can be obtained by using these detectors in combination.

Preparation of Working Electrodes. A glassy carbon electrode (5 mm in diameter) was used as working electrode and was polished with 0.05 μm Al_2O_3 powder before use. For preparation of catalyst inks, PtCu and PtCuNi catalysts were mixed with carbon (Vulcan XC-72) with mass ratio of 1:4 and then dissolved in isopropanol/water mix solvents. For Pt/C catalyst (20 wt.% Pt, Johnson Matthey), the catalysts were dissolved in isopropanol/water mix solvents. The concentration of the catalyst inks were $1mg_{metal}$/ml~$2mg_{metal}$/ml. After that, the catalyst inks were sonicated for 30 min. For preparation of working electrodes, 10μL inks were deposited on glassy carbon electrode and dried to form a uniform thin film that was further characterized in electrochemical cell.

Electrochemical Measurements. All electrochemical tests were performed in a standard three-electrode electrochemical cell with Pt foil (1 cm^2) and saturated Ag/AgCl electrode was used as

counter electrode and reference electrode at room temperature (~25 °C) using a Princeton P4000 electrochemical workstation. The working electrode was first subjected to CV treatment (0.05V-1.25V) for 50 cycles to clean the surface ligands. The CO stripping experiments were conducted in 0.5 M H_2SO_4. The potential was first held at 0.08 V vs RHE for 10 min in CO-saturated solutions in order to allow sufficient CO adsorption onto the surface of catalysts. Subsequently, the solution was deaerated with N_2 for 20 min to remove freely dissolved CO molecules. Methanol oxidation reaction (MOR) was carried out in a solution containing 0.5 M H_2SO_4 and 1 M CH_3OH. The working electrodes were first subjected to CV treatment in 0.5 M H_2SO_4 to reach a stable state and then formic acid oxidation tests were performed. Oxygen reduction reaction (ORR) polarization curves was recorded at room temperature in an O_2 -saturated 0.1 M $HClO_4$ aqueous solution with a sweep rate of 20 mV/s and a rotation rate of 1600 rpm. The stability tests were carried out by 5,000 cycles between 0.6 ~ 1.0 V.

Supplementary Figures

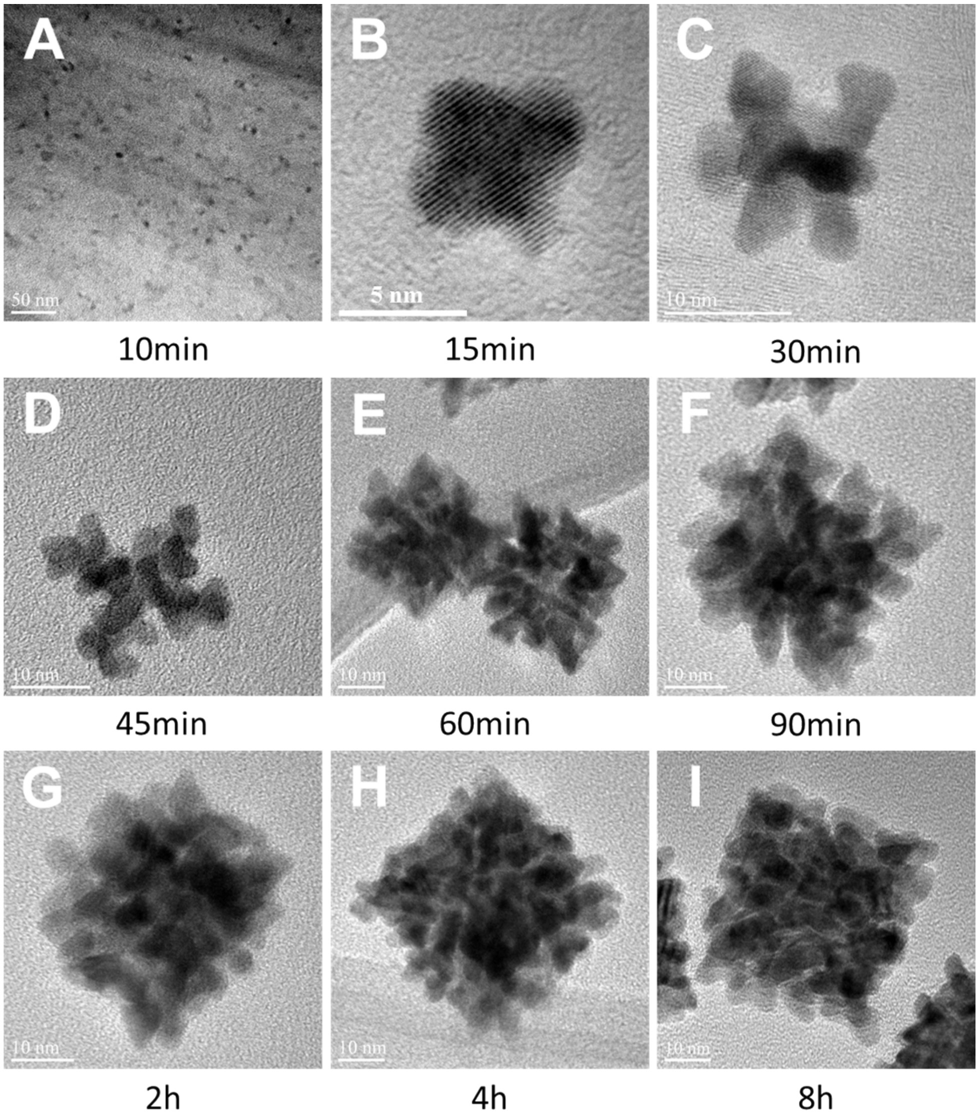

Fig. S1 (A)–(I) Time dependent morphology evolution of dendritic Pt_3Cu cubes.

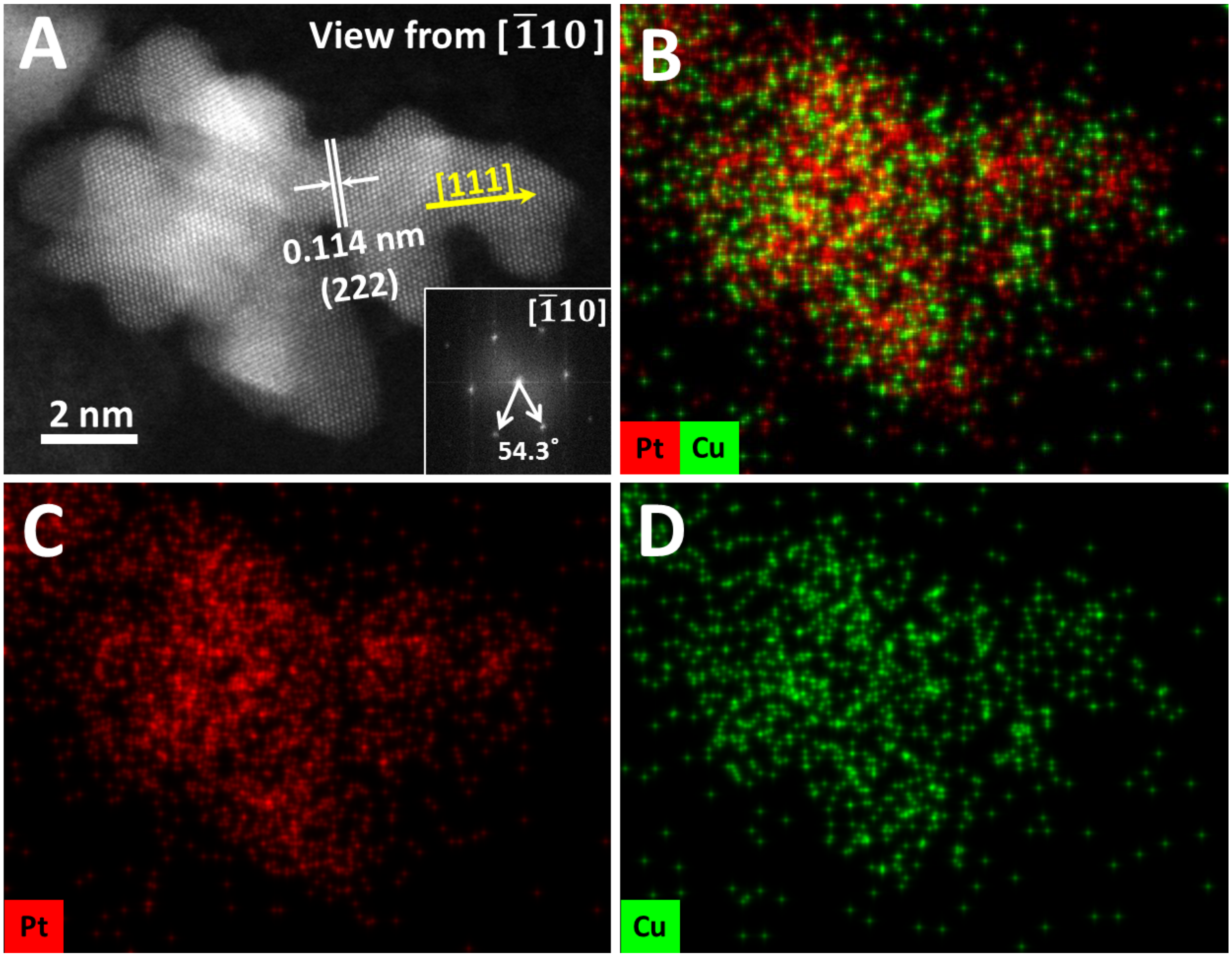

Fig. S2 (A) HAADF-STEM image of the intermediate, revealing [111] growth direction of the branches. (B)-(D) EDX-Mapping revealed alloyed structure of the intermediate. Pt : Cu = 75.42% : 24.58% = 3.07 : 1

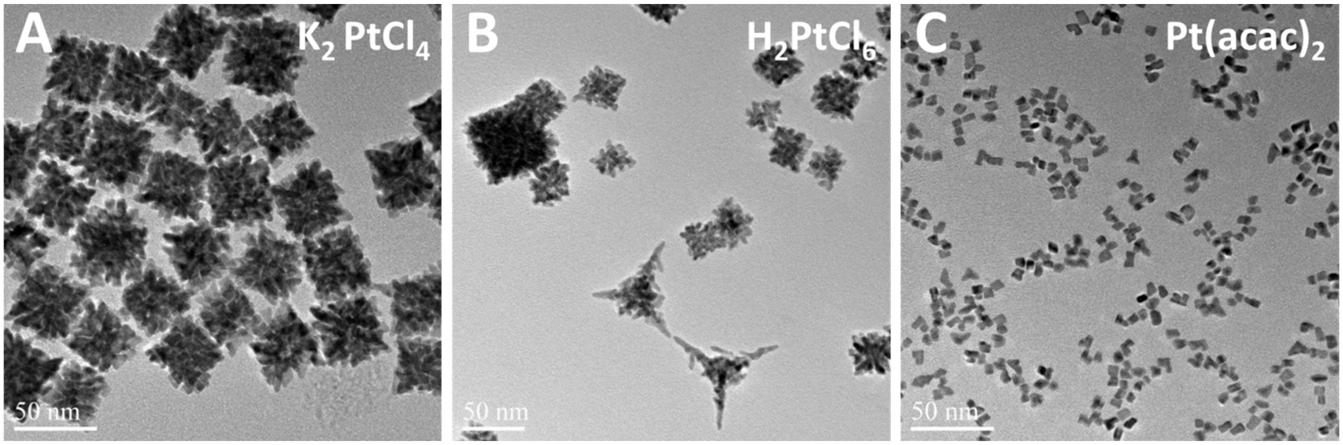

Fig. S3 Influence of Pt precursor species on the formation of dendritic PtCu cubes

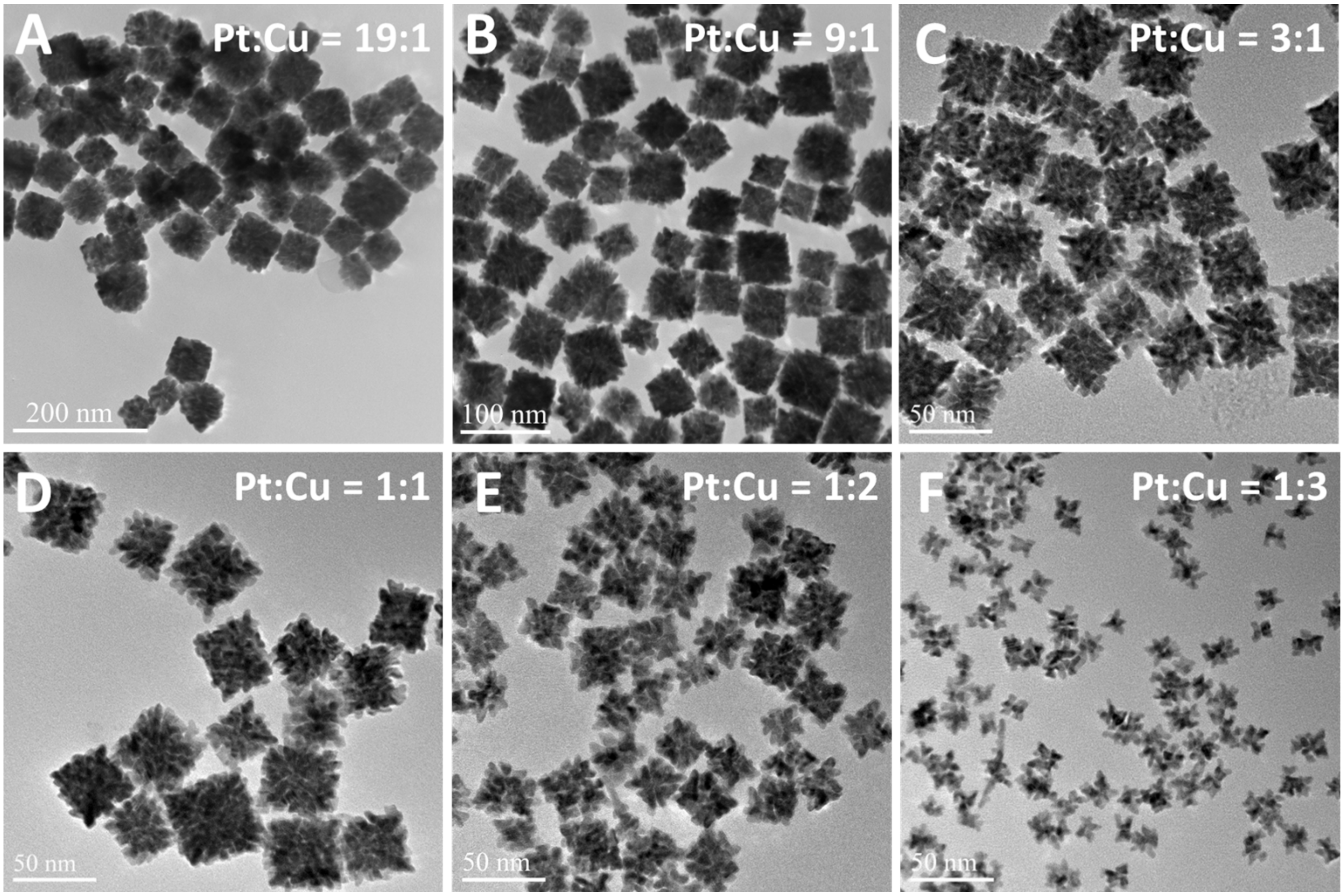

Fig. S4 Influence of precursor ratio on the formation of dendritic PtCu cubes

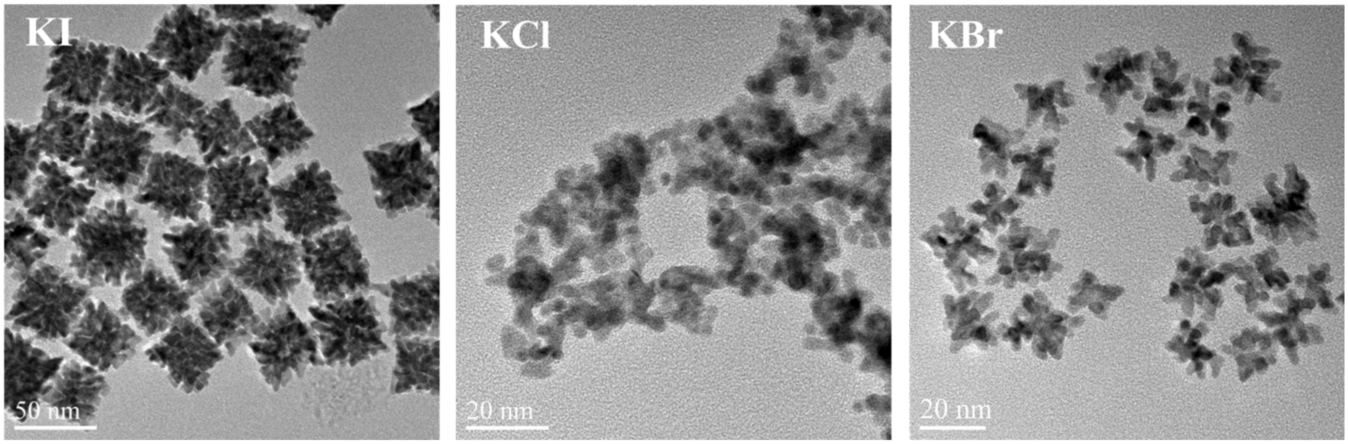

Fig. S5 Influence of coordination reagents on the formation of dendritic PtCu cubes

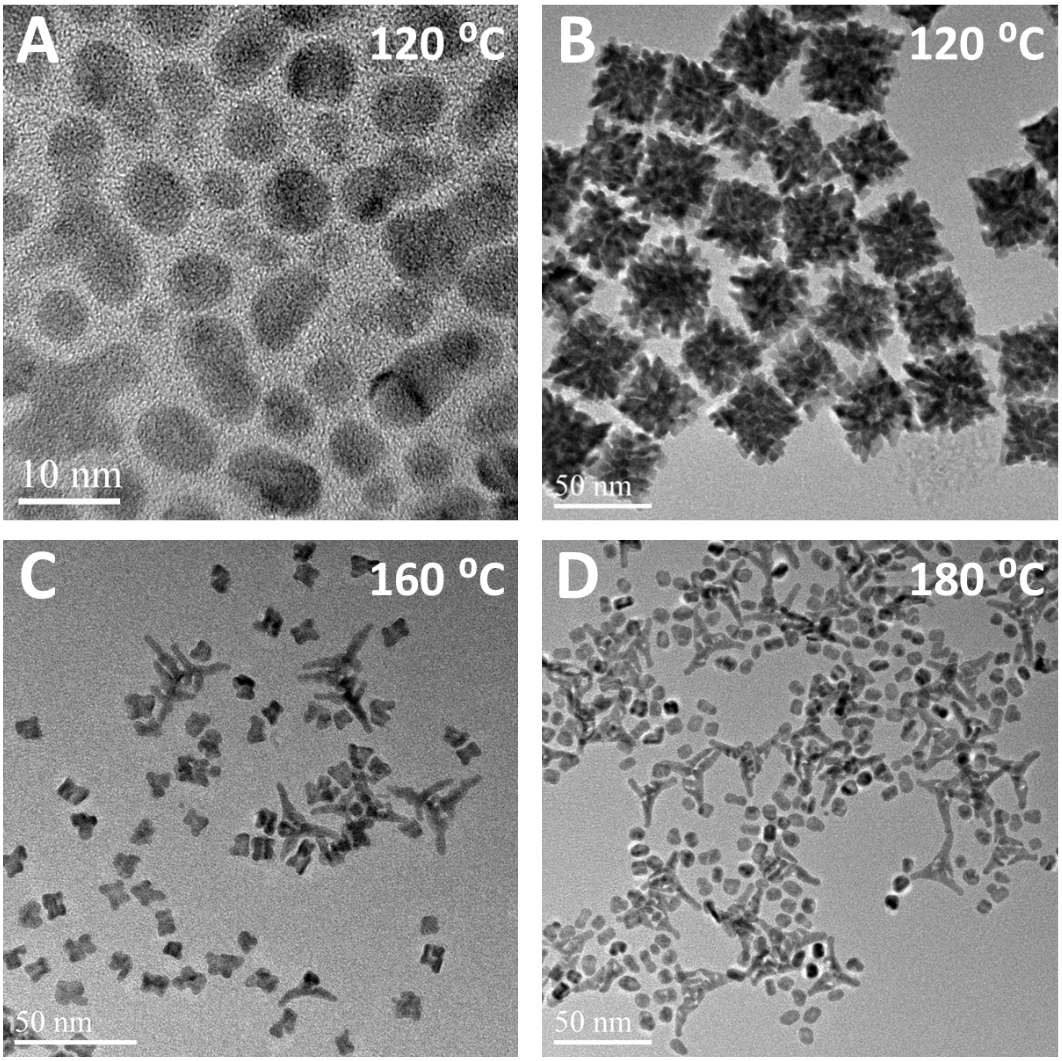

Fig. S6 Influence of reaction temperature on the formation of dendritic PtCu cubes

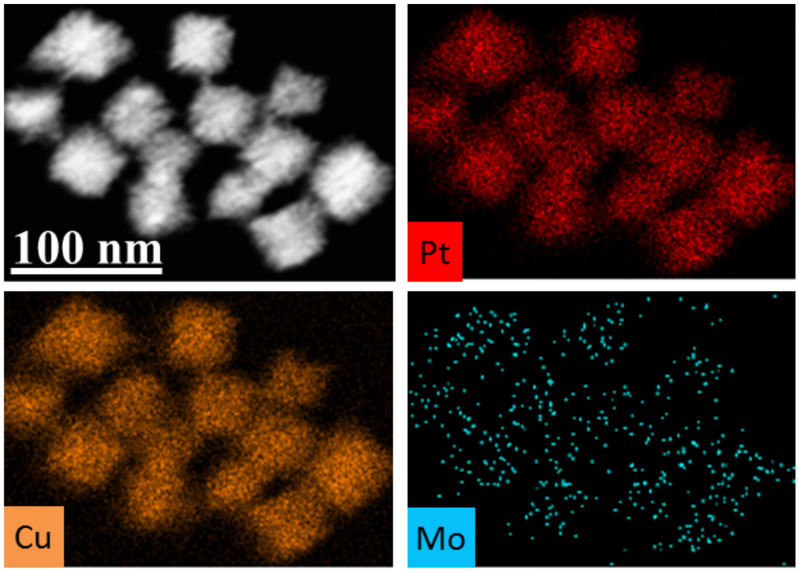

Fig. S7 HAADF-STEM image and EDS-Mapping of PtCuMo nanocubes

Table S1. Composition variation of the PtCuNi cubes with different feeding ratio

Pt : Cu : Ni	
**Feeding Ratio**	**Composition**
9 : 1 : 1	8.65 : 1 : 0.55
9 : 1 : 3	8.73 : 1 : 1.20
9 : 1 : 5	8.49 : 1 : 2.78
9 : 1 : 7	8.11 : 1 : 4.11
9 : 1 : 9	8.55 : 1 : 5.65

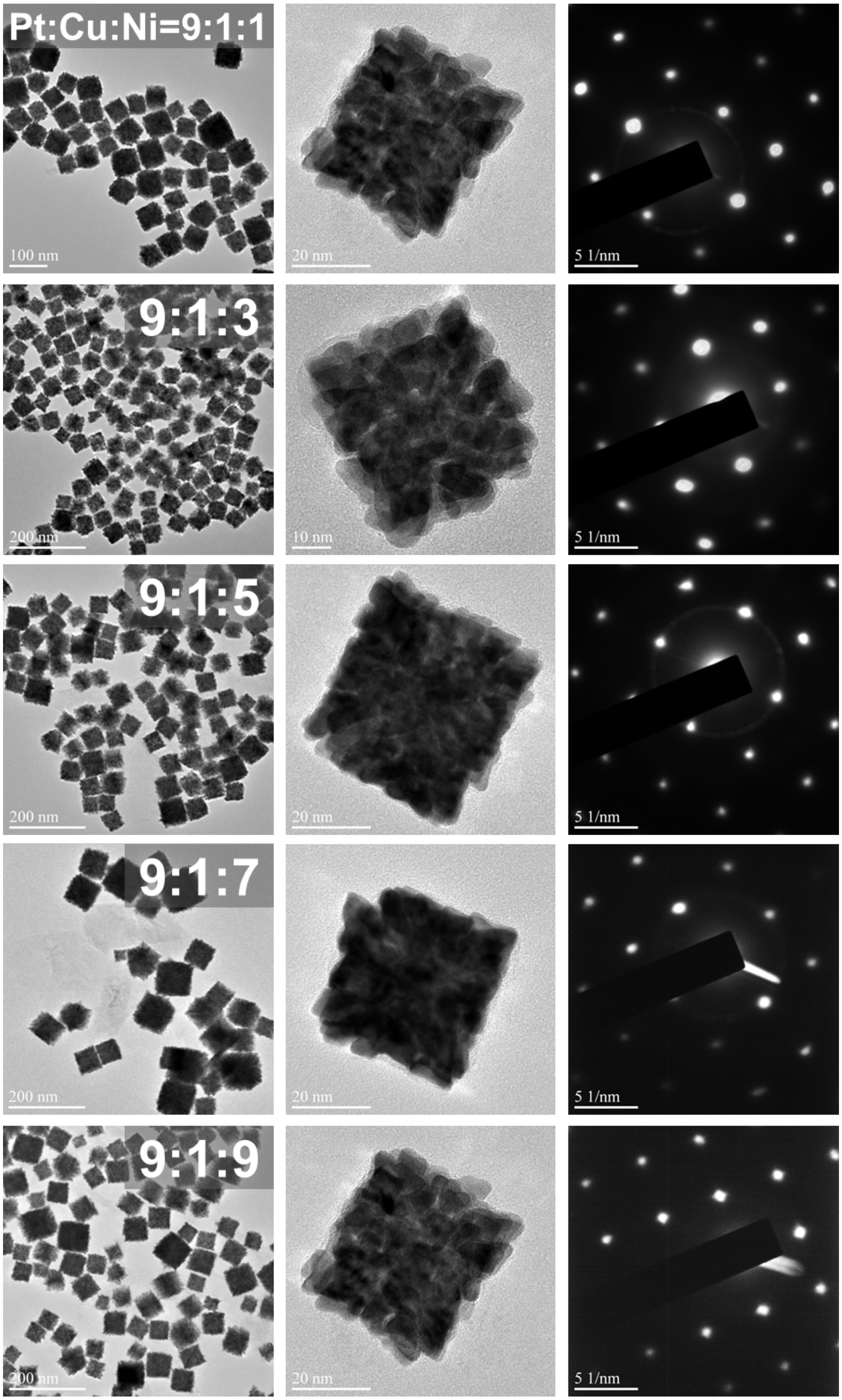

Fig. S8 TEM images and corresponding electron diffraction patterns of PtCuNi samples with different feeding ratios

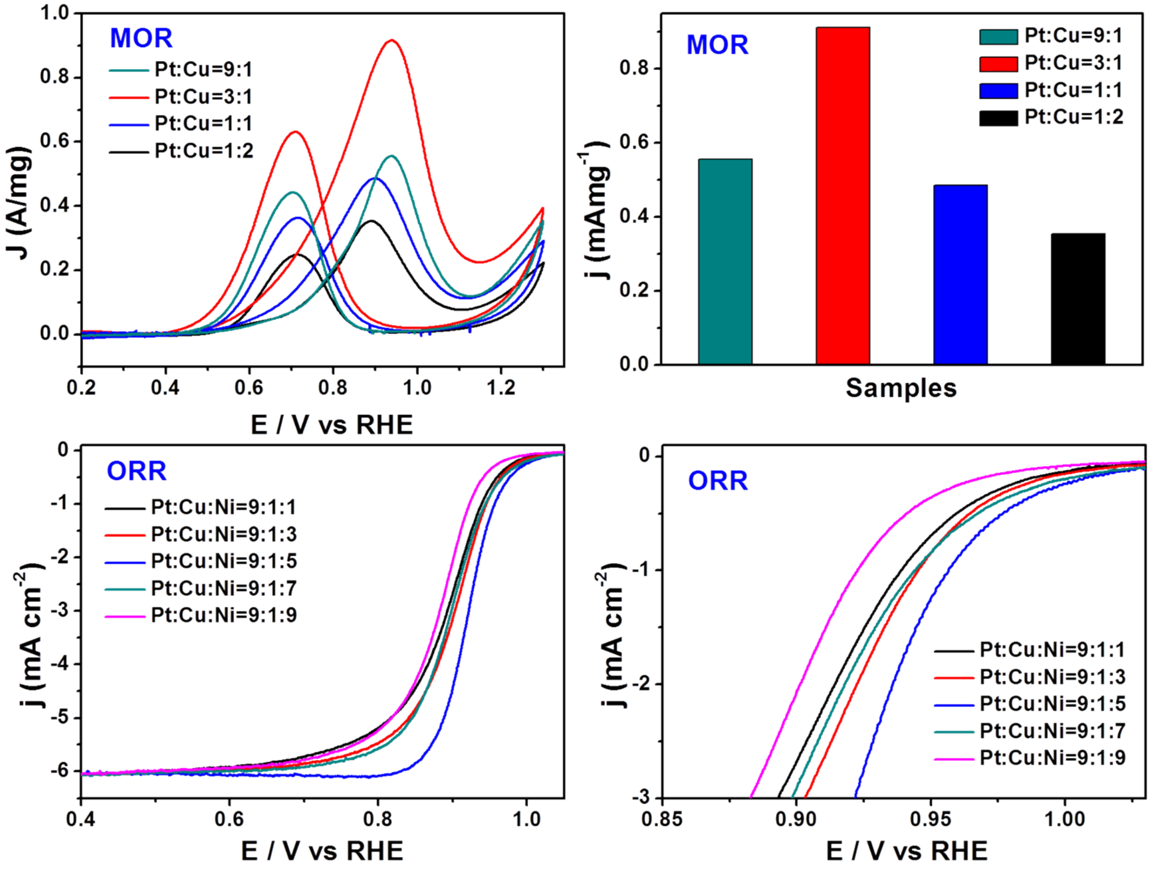

Fig. S9 Elecrtocatalytic methanol oxidation reaction (MOR) and oxygen reduction reaction performances of PtCu and PtCuNi samples with different synthetic feeding ratios

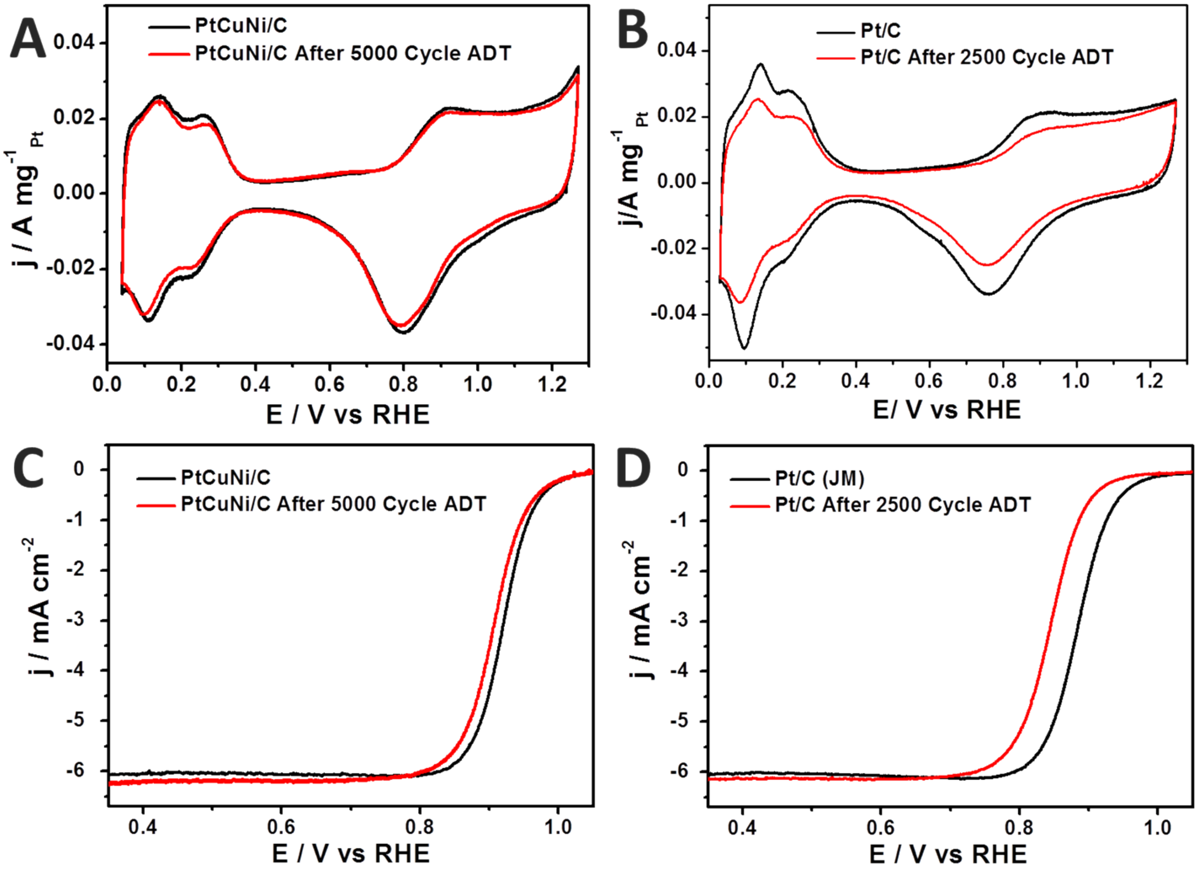

Fig. S10 (A), (B) CV curves and (C), (D) ORR polarization curves of PtCuNi/C and PtC before and after 5000/2500 cycles of oxygen reduction reaction (ORR) accelerated durability test (ADT). ADT was performed in O_2-saturated 0.1 M $HClO_4$ solution with a sweep rate of 50 mV/s.

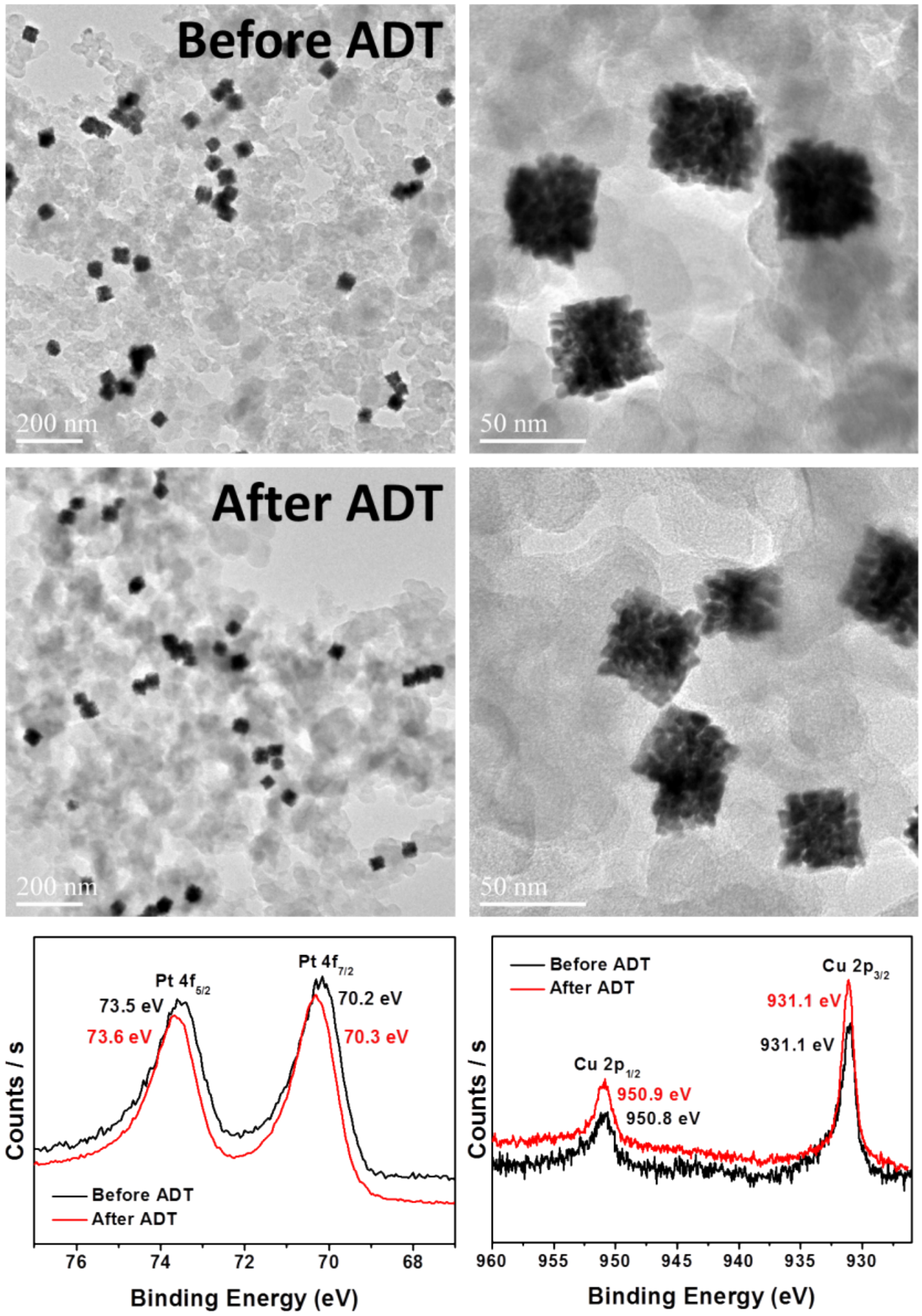

Fig. S11 HRTEM images and XPS spectra of PtCuNi/C before and after 5000 cycles of oxygen reduction reaction (ORR) accelerated durability test (ADT). ADT was performed in O_2-saturated 0.1 M $HClO_4$ solution with a sweep rate of 50 mV/s.
